# Inclusion of sprints in moderate intensity continuous training leads to muscle oxidative adaptations in trained individuals

**DOI:** 10.14814/phy2.13976

**Published:** 2019-02-21

**Authors:** Thomas P. Gunnarsson, Nina Brandt, Matteo Fiorenza, Morten Hostrup, Henriette Pilegaard, Jens Bangsbo

**Affiliations:** ^1^ Department of Nutrition, Exercise and Sports University of Copenhagen Copenhagen Denmark; ^2^ Department of Biology University of Copenhagen Copenhagen Denmark

**Keywords:** High‐intensity interval training (HIIT), human performance, metabolic stress, PGC‐1a mRNA, skeletal muscle single fibers

## Abstract

This study examined adaptations in muscle oxidative capacity and exercise performance induced by two work‐ and duration‐matched exercise protocols eliciting different muscle metabolic perturbations in trained individuals. Thirteen male subjects ($\dot{V}$O_2_‐max 53.5 ± 7.0 mL·kg^−1^·min^−1^) (means ± SD) performed 8 weeks (three sessions/week) of training consisting of 60 min of moderate intensity continuous cycling (157 ± 20 W) either without (C) or with (C+S) inclusion of 30‐s sprints (473 ± 79 W) every 10 min. Total work performed during training was matched between groups. Muscle biopsies and arm venous blood were collected before as well as immediately and 2 h after exercise during the first and last training session. Plasma epinephrine and lactate concentrations after the first and last training session were 2–3‐fold higher in C+S than in C. After the first and last training session, muscle phosphocreatine and pH were lower (12–25 mmol·kg d.w.^−1^ and 0.2–0.4 units, respectively) and muscle lactate higher (48‐64 mmol·kg d.w.^−1^) in C+S than in C, whereas exercise‐induced changes in muscle PGC‐1*α* mRNA levels were similar within‐ and between‐groups. Muscle content of cytochrome c oxidase IV and citrate synthase (CS) increased more in C+S than in C, and content of CS in type II muscle fibers increased in C+S only (9–17%), with no difference between groups. Performance during a 45‐min time‐trial improved by 4 ± 3 and 9 ± 3% in C+S and C, respectively, whereas peak power output at exhaustion during an incremental test increased by 3 ± 3% in C+S only, with no difference between groups. In conclusion, addition of sprints in moderate intensity continuous exercise causes muscle oxidative adaptations in trained male individuals which appear to be independent of the exercise‐induced PGC‐1*α* mRNA response. Interestingly, time‐trial performance improved similarly between groups, suggesting that changes in content of mitochondrial proteins are of less importance for endurance performance in trained males.

## Introduction

Moderate intensity continuous (MICT) and high‐intensity interval (HIIT) training increase content of muscle mitochondrial enzymes in skeletal muscle of untrained and recreationally active individuals, which is coinciding with enhanced endurance performance (Holloszy 1967; Wenger and Bell 1986; MacDougall et al. 1998; Burgomaster et al. 2005, 2006). The importance of intensity, work performed, and duration for adaptations in mitochondrial enzymes and endurance performance have been examined in studies comparing work‐ and duration‐matched MICT versus HIIT (Henriksson and Reitman 1976; Daussin et al. 2008; Granata et al. 2016; MacInnis et al. 2017). However, the effect of inclusion of sprint exercise on the training response to MICT has not been investigated. Sprint interval training “SIT” and speed endurance training “SET,” where sprints of typically 30‐s duration at near maximal intensity interspersed with 2–4 min recovery periods are performed, have been shown to effectively enhance performance in trained individuals and content of mitochondrial enzymes in untrained individuals (Hostrup & Bangsbo., 2017 MacInnis et al. 2017). Thus, it may well be that inclusion of 30‐s sprints in MICT augments training‐induced adaptations in mitochondrial enzymes and performance. Nevertheless, the effect of inclusion of 30‐s sprints on training‐induced adaptations to MICT in muscle oxidative enzymes and performance has not been examined in a controlled work‐ and duration‐matched design in trained individuals.

Training‐induced adaptations in muscle mitochondria are promoted at the molecular level through activation of a variety of intracellular signaling pathways regulating mRNA transcription of genes encoding mitochondrial proteins (Egan et al. 2010; Perry et al. 2010). The transcriptional coactivator peroxisome proliferator‐activated receptor‐ϒ coactivator‐1*α* (PGC‐1*α*) is believed to be a key mediator of the exercise‐induced transcriptional regulation of genes encoding mitochondrial proteins (Puigserver et al. 1998). Despite robust effects toward an oxidative skeletal muscle phenotype, PGC‐1*α* may not be necessary for the majority of oxidative adaptations associated with exercise training (Egan and Zierath 2013). The exercise‐induced response in PGC‐1*α* mRNA levels has been shown to be regulated in an intensity dependent manner (Egan et al. 2010), which is likely associated with exercise‐induced muscle metabolic perturbations. Nevertheless, studies have shown similar exercise‐induced PGC‐1*α* mRNA responses during work‐matched exercise protocols differing in metabolic perturbations (Bartlett et al. 2012; Brandt et al. 2016).

Mitochondrial content has been shown to increase similarly in response to SET (30‐s all‐out sprints) and MICT in untrained and recreationally active individuals despite an exercise duration ratio of ~30:1 in favor of MICT (Gibala et al. 2006; Burgomaster et al. 2008) suggesting that intensity is more important than total work performed. Given the suggested key role of PGC‐1*α* in regulation of mitochondrial biogenesis (Puigserver et al. 1998) and that exercise‐induced PGC‐1*α* mRNA response has been shown to be higher when combining MICT and SET compared to MICT and SET alone (Skovgaard et al. 2016), chronic repetitions of SET in combination with MICT may lead to superior mitochondrial adaptations compared to MICT alone.

Only a few studies have compared work‐matched moderate intensity continuous and interval‐based exercise interventions on mitochondrial adaptations (Henriksson and Reitman 1976; Daussin et al. 2008; Granata et al. 2016; MacInnis et al. 2017). Granata et al. (2016) is, to our knowledge, the only study to compare SET and MICT on mitochondrial adaptations and performance observing superior adaptations in PGC‐1*α* protein content and mitochondrial specific respiration following 4 weeks of SET. However, SET and MICT were not work‐matched and whether oxidative adaptations to work‐matched SET and MICT affect performance differently than low‐volume SET remains unknown. Mitochondrial adaptations have almost exclusively been examined in mixed muscle homogenate of humans (Henriksson and Reitman 1976; Howald et al. 1985). Single fiber analysis allows for assessment of fiber‐type specific adaptations in oxidative proteins (Christensen et al. 2015). MICT was shown to elicit a more marked increase in mitochondrial content in type II than in type I muscle fibers (Howald et al. 1985), whereas no fiber‐type differences were observed between work‐matched MICT and HIIT (MacInnis et al. 2017). Whether oxidative adaptations can be expected when sprints are included in MICT is unknown, but given the intensity during sprinting, a greater recruitment of type II fibers can be expected (Krustrup et al. 2004), inducing more pronounced oxidative adaptations in these fibers. Whether to expect oxidative adaptations in type I fibers is unclear as a larger abundance of oxidative markers have been observed in these fibers (Howald et al. 1985; Thomassen et al. 2013; Christensen et al. 2015) and no changes in oxidative capacity in a fiber‐type dependent manner have been observed following SET in trained individuals (Christensen et al. 2015; Nyberg et al. 2016; Skovgaard et al. 2018). Thus, the aim of this study was to compare the effect of MICT without or with inclusion of repeated 30‐s sprints on adaptations in mitochondrial proteins in type I and II muscle fibers, as well as on cycling performance. We hypothesized that addition of 30‐s sprints in MICT would lead to superior adaptations in performance and mitochondrial enzymes in mixed muscle of trained individuals as well as in a fiber‐type dependent manner compared to MICT alone.

## Methods

### Subjects

Fourteen healthy, nonsmoking, physically active male subjects volunteered to participate in the study. However, samples from only twelve (all muscle data) and thirteen (performance data) subjects are included in the data analysis as one subject lacked biopsy material to complete all the analysis and one subject did not complete post testing due to personal problems. The average age of the subjects was 25 years (range: 19–34 year) and the average BMI was 23.9 kg·m^−2^ (range: 17.5–29.3 kg·m^−2^). Subject characteristics are presented in Table 1. Subjects were engaged in 1–3 weekly training sessions (team sports, endurance and/or strength training) but were not involved in regular competitions. Subjects were fully informed of experimental procedures and written consent was obtained prior to the study. Study procedures were approved by the Ethics Committee of Copenhagen and Frederiksberg communities and conformed to the Code of Ethics of the World Medical Association (Declaration of Helsinki, Tokyo, 2008).

**Table 1:** Subject characteristics before an 8‐week intervention period consisting of 60 min of continuous moderate intensity cycling exercise without (C; *n* = 7) or with (C+S; *n* = 6) inclusion of sprints

	C	C+S
Age (years)	25.6 ± 5.4	24.7 ± 2.7
Weight (kg)	79.3 ± 15.1	79.8 ± 4.2
Maximal oxygen uptake (mL·min^−1^)	4202 ± 469	4211 ± 556
Maximal oxygen uptake (mL·min^−1^·kg^−1^)	53.8 ± 5.5	53.1 ± 9.1

Values are presented as means ± SD.

### Study design

These data are secondary analyses of a larger previously published study, focusing on exercise and exercise training‐induced increase in autophagy markers in trained human skeletal muscle (Brandt et al. 2018).

Prior to the study, subjects underwent an incremental test to exhaustion on an electronically braked cycle ergometer (Monarch 839E, Vansbro, Sweden) to ensure that they had a maximal oxygen uptake ($\dot{V}$O_2_‐max) above 45 mL·min^−1^·kg^−1^. The subjects cycled for 5 min at 125 W and 200 W followed by an incremental test to exhaustion (increase in workload of 25 W·min^−1^) until volitional exhaustion defined as pedaling frequency dropping below 50 rpm. The peak power output achieved during an incremental test to exhaustion was noted. Pulmonary oxygen uptake was measured (Oxycon Pro, Viasys Healthcare, Hoechberg, Germany) during the test and $\dot{V}$O_2_‐max was determined as the highest value achieved during any 30‐s period. Criteria used for achievement of $\dot{V}$O_2_‐max were a plateau in $\dot{V}$O_2_ despite an increased workload and a respiratory exchange ratio above 1.10. $\dot{V}$O_2_ during submaximal and maximal cycling was used to calculate individual workloads on experimental days (60% of $\dot{V}$O_2_‐max). In addition, subjects completed a 45‐min time‐trial performance test (TT). Based on $\dot{V}$O_2_‐max and TT rank scores, subjects were matched in pairs (1 to 14) and randomly assigned pairwise to moderate intensity continuous training groups without (C) or with (C+S) inclusion of sprint intervals for an 8‐week intervention period with three training session per week.

### Experimental days

The subjects conducted three experimental days before and after the 8‐week intervention period. Order of experimental days A and B was randomized between subjects with the order being the same before and after the intervention period. Experimental day C was conducted during the first and last training sessions of the 8‐week intervention period.

#### Experimental day A

Subjects completed two 5‐min cycling bouts at 123 ± 2 and 198 ± 2 W interspersed by 5 min of recovery. Immediately after completion of the second bout, subjects completed an incremental test to exhaustion with a 25 W·min^−1^ increase in workload until voluntary exhaustion or until pedaling frequency dropped below 50 RPM. Peak power output achieved at exhaustion during the incremental test to exhaustion was noted and pulmonary $\dot{V}$O_2_ measured as described above.

#### Experimental day B

After a 5‐min warm‐up period at a work load eliciting 60% of $\dot{V}$O_2_‐max (157 ± 23 W) followed by 2 min of rest, the subjects completed a 45‐min TT. Throughout the TT, load and cadence were controlled by the subjects, who were blinded to the total amount of work completed. Heart rate (Polar team system, Polar, Electro Oy) was sampled continuously and water was allowed ad libitum during the test.

#### Experimental day C (first and last training session)

Subjects reported to the laboratory in the morning (between 8 and 9 a.m.) 2 h after consumption of a self‐chosen breakfast, which was replicated during post testing. Within 60 min of arrival, a catheter (18 gauge, 32 mm) was inserted in an antecubital vein allowing blood sampling. After catheterization, local anesthesia (1 ml of lidocaine, 20 mg·mL^−1^ without adrenaline) was administered to the skin on m. vastus lateralis and two incisions (~2 mm) were made and a muscle biopsy at rest was obtained from the most distal incision. Then subjects completed their first training session consisting of 60 min of cycling with or without sprints as described above. Heart rate (Polar team system, Polar, Electro Oy) and $\dot{V}$O_2_ (Oxycon Pro, Viasys Healthcare, Hoechberg, Germany) were measured during the session. Immediately at cessation of exercise, a muscle biopsy was taken (0 h) from the proximal incision, thereafter the subjects were put in the supine position on a bed. After 2 h of resting, an additional muscle biopsy was taken from the opposite leg. The muscle samples were immediately frozen in liquid nitrogen and stored at −80°C until the analysis. A blood sample was collected before (rest), after 20, 40, and 60 min of exercise as well as 3, 10, and 120 min after exercise. The subjects were allowed to ingest water ad libitum but no foods during the experimental day.

### Training

During each training session, the continuous training group without sprints (C; *n* = 7) cycled on an electronically braked cycle ergometer (Monarch 839E, Vansbro, Sweden) for 60 min at a constant power output (157 ± 20 W) eliciting ~60% of pre‐intervention $\dot{V}$O_2_‐max. The continuous training group with sprints (C+S; *n* = 6) cycled at an average power output of 158 ± 26 W and performed six repeated 30‐s sprints (473 ± 79 W) every 10 min followed by a reduced power output (102 ± 17 W) for 3:24 (min:s) eliciting ˜60% of pre‐intervention $\dot{V}$O_2_‐max. The work load was matched between the first and last training session within groups.

### Muscle analyses

A part (~20 mg) of the muscle sample was saved separately for mRNA analysis. Another part (~80 mg) of the muscle sample was freeze dried for 48 h and dissected free of all nonmuscle elements. Dissection was performed under a stereo microscope with an ambient temperature of ~18°C and a relative humidity below 30%. After dissection, muscle tissue was weighed and separated for subsequent analyses.

#### RNA isolation, reverse transcription, and real‐time PCR

Total RNA was isolated from ~20 mg muscle tissue (w.w.) by a modified guanidinium thiocyanate‐phenol‐chloroform extraction method from Chomczynski and Sacchi (Chomczynski and Sacchi 1987). Superscript II RNase H‐system and Oligo dT (Invitrogen, Carlsbad, CA, USA) were used to reverse transcribe the mRNA to cDNA as described previously (Chomczynski and Sacchi 1987) except for the use of a TissueLyser (TissueLyser II, Qiagen, Valencia, CA, USA) for homogenization. Quantification of cDNA as a measure of mRNA content of a given gene was performed by real‐time PCR using an ABI 7900 sequence‐detection system (Applied Biosystems, Foster City, CA, USA). Primers and TaqMan probes were designed from human specific databases from ensemble (www.ensembl.org/Homo_sapiens/Info/Index) and Primer Express (Applied Biosystems) (Table 2). Self‐designed TaqMan probes were labeled with 5′‐6‐carboxyfluorescein (FAM) and 3′‐6‐carboxy‐N,N,N′,N′‐tetramethylrhodamine (TAMRA) and obtained from TAG Copenhagen (Copenhagen, Denmark). Cyclophilin A mRNA was amplified using a 5`‐VIC‐ and 3′‐TAMRA‐labeled predeveloped assay reagent (Applied Biosystems). Cyclophilin A was used as endogenous control (Murphy et al. 2003, 2004). Real‐time PCR was performed (in triplicates) in a total reaction volume of 10 *μ*L using Universal Mastermix with UNG (Applied Biosystems). The obtained cycle threshold values reflecting the initial content of the specific transcript in the samples were converted to a relative amount by using standard curves constructed from serial dilution of a pooled sample made from all samples. For each cDNA sample, the amount of the given target was normalized to Cyclophilin A mRNA.

**Table 2:** Primers and TaqMan probe sequences used for real‐time PCR. Peroxisome proliferator‐activated receptor‐ϒ coactivator‐1*α* (PGC‐1*α*)

	PGC‐1*α* mRNA
Forward primer	5′‐CAAGCCAAACCAACAACTTTATCTCT‐3′
Reverse primer	5′‐CACACTTAAGGTGCGTTCAATAGTC‐3′
TaqMan Probe	5′‐AGTCACCAAATGACCCCAAGGGTTCC‐3′

#### Protein expression in muscle lysates

A part of the muscle sample (~5 mg d.w.) was split in two for double protein determination and then homogenized and centrifuged to exclude non dissolved structures as previously described (Bangsbo et al. 2009). Protein concentration was determined in lysates using BSA standards (Pierce Biotechnology, Inc., Rockford, IL), and lysates were diluted to appropriate protein concentrations with a 6x Laemmli sample buffer and ddH_2_O. For the determination of protein expression by the method of western blotting, equal amounts of total protein (5–15 *μ*g in accordance with the antibody optimization) were loaded on 4–15% precasted stain‐free Tris‐HCl gels (Bio‐Rad Laboratories, Hercules, CA). Muscle homogenate lysate proteins were separated by SDS‐PAGE (55 mA per gel and maximum 150 V) for ~90 min and semidry transferred to a PVDF membrane (Millipore A/S, Copenhagen, Denmark) for 120 min at 70 mA per gel and at a maximum of 25 V. After protein transfer, membranes were incubated overnight with a primary antibody diluted in either 2% nonfat milk COX IV (~14 kDa), 1:500 dilution (Sc‐58348, Santa Cruz Biotechnology), *β*‐HAD (83 kDa), 1:1000 dilution (ab54477, Abcam)], or 3% BSA [CS (48 kDa), 1:3000 dilution (ab96600, Abcam), PFK (85 kDa), 1:2000 dilution (Sc‐166722, Santa Cruz Biotechnology)]. Afterward, membranes were incubated with secondary horseradish peroxidase‐conjugated antibodies diluted 1:5000 in 2% nonfat milk or 3% BSA depending on the primary antibody (P‐0447, P‐0448, and P‐0449, DakoCytomation) for ~1 h at room temperature. The membrane staining was visualized by incubation with a chemiluminescent horseradish peroxidase substrate (Merck Millipore) prior to image digitalization on a Chemi Doc MP (Bio‐Rad Laboratories, Hercules, CA). Net band intensities were quantified using Image Lab (Image Lab v. 4.0, Bio‐Rad Laboratories) and the average signal intensity of the two adherent samples was used as result for individual time points.

#### Protein expression in single muscle fibers

For each subject, 30–60 segments of single fibers were dissected from the freeze‐dried muscle biopsies. Fiber length was determined (1.7 ± 0.7 mm, *n* = 1008) under a light microscope and stored in single microfuge tubes. Twenty microliters of 6x Laemmli buffer (0.7 mL of 0.5 mol/L Tris base, 3 mL glycerol, 0.93 g DTT, 1 g SDS and 1.2 mg Bromophenol Blue), diluted (1:3), was added to each tube and incubated for 1 h at room temperature. A part of each single fiber segment was analyzed for MHCI, MHCII, and SERCA1 content by the method of western blotting, and determined as being a type I or II muscle fiber. If a fiber segment showed bands for both MHCI and MHCII/SERCA1, it was discarded from the analysis. In order to determine the fiber type of each of the single fiber segments, 5 *μ*L of the sample was loaded on a 26‐well Tris‐Tricine 4–15% Criterion gel (Bio‐Rad Laboratories, Solna, Sweden) and by the method of western blotting characterized as either a type I or type II muscle fiber using antibodies specific for myosin heavy chain type I and type II as well as the FT‐specific SERCA1 protein (Thomassen et al. 2013). Antibodies were mouse monoclonal IgM, A4.840 (0.5 *μ*g·mL^−1^), Developmental Studies Hybridoma Bank (DSHB), University of Iowa, USA for ST fibers and mouse monoclonal IgG, A4.74 (2 *μ*g·mL^−1^), DSHB, for FT fibers both developed by Dr. Blau and SERCA1 mouse monoclonal, MA3‐912 (0.1 *μ*g·mL^−1^), Thermo Scientific.

Following fiber type determination of each single fiber, 15 *μ*L was loaded onto additional gels to determine the expression of CS and PFK in single fibers using the method of western blotting. In short, 20 fibers from each subject (five type I and five type II fibers from before and after the intervention period), matched for length, were loaded (~15 *μ*L) on a 26‐well 4–15% precasted stain‐free Tris‐HCl gel (Bio‐Rad Laboratories, Hercules, CA), together with a 3 point standard curve (0.5, 1.0 and 2.0 *μ*g·*μ*L^−1^). A stain‐free image was obtained after a 5 min incubation period (light exposure) using a Chemi Doc MP station (Bio‐Rad Laboratories, Hercules, CA). Membranes were incubated overnight with ~20 mL of primary antibody diluted in 3% BSA [CS (~48 kDa), 1:3000 dilution (ab96600, Abcam), PFK (~85 kDa), 1:2000 dilution (Sc‐166722, Santa Cruz Biotechnology)]. Membranes were visualized using chemiluminescent HPS SuperSignal West Femto (Thermo Scientific).

Protein content of each single fiber was related to the respective total protein content from the stain‐free imaging. In order to compare fibers loaded on different gels, single values were normalized to the mean of all before training type I fibers.

#### Muscle metabolites

A part of the muscle sample (~2 mg d.w.) was extracted in 3 mol/L perchloric acid (PCA). The supernatant was neutralized to pH 7.0 with 2 mol/L KHCO_3_ and analyzed for PCr and lactate using fluorometric methods (Fluoroscan Ascent, Thermo Scientific, Waltham, MA) (Lowry and Passonneau 1972). Another part of the muscle sample (~1 mg of d.w.) was homogenized in a 120 *μ*L nonbuffering solution containing 145 mmol/L KCl, 10 mM NaCl, and 5 mmol/L iodoacetic acid, allowing for muscle pH measures (Radiometer GK2801). A part of the muscle sample (~2 mg d.w.) was extracted in 1 mol/L HCl and hydrolyzed at 100°C for 3 h allowing determination of muscle glycogen by the hexokinase method (Lowry and Passonneau 1972).

#### Maximal muscle enzyme activity

A part of the muscle (~2 mg of d.w.) was homogenized (1:400) in a 0.3 mol/L phosphate BSA buffer adjusted to pH 7.7 and analyzed for the maximal enzyme activity of phosphofructokinase (PFK), hydroxyacyl‐CoA dehydrogenase (HAD), and citrate synthase (CS) using fluorometric methods (Fluoroscan Ascent, Thermo Scientific, Waltham, MA) (Lowry and Passonneau 1972).

### Blood analyses

Whole blood samples were drawn in heparinized 2 mL syringes and stored on ice until analysis for blood lactate (ABL 800 Flex, Radiometer, Copenhagen) and plasma FFA (Hitachi 912 Automatic Analyzer, Roche Diagnostic). Catecholamine samples were drawn in dry 5 mL syringes and transferred to Eppendorf tubes containing 30 *μ*L EDTA (0.2 mol/L). The whole blood was initially spun at 20,000*g* for 2 min and pipetted plasma was stored at −20°C until further analysis (Plasma ELISA High Sensitive kit, LDN, Nordhorn, Germany).

### Statistics

Statistical calculations were performed using SigmaPlot Version 13.0. Values are presented as means±SD unless stated otherwise. Sample power analysis was performed prior to the study based on expected changes (~10% with a SD ~5%) in CS protein content with a power level of 0.8, and *α *= 0.05 giving a minimum required sample size of *n* = 6 per group. Changes of ~10% from pre levels were based on half the change in CS protein content as reported by Little et al. (2010) as subjects in this study had ~20% higher $\dot{V}$O_2_‐max, and it is well recognized that the muscle mitochondrial adaptive response is reduced in muscle accustomed to regular MICT. Thus, the expected effect size of C+S was 10% compared to C. Based on previous experiments we expected a dropout rate of up to 15%, thus recruiting a total of 14 subjects. Physiological, metabolic and mRNA, maximal enzyme activity, ventilation, and performance data in response to training were evaluated using a two‐way analysis of variance (ANOVA) for repeated measures. If a main effect was observed, a Student–Newman–Keul`s post hoc test was used to detect within time and between group differences. Relative changes in protein expression (muscle homogenates and single muscle fibers) within groups were examined using a one sample t‐test. A significance level of *P* < 0.05 was chosen.

## Results

### Performance and maximal oxygen uptake

**Figure 1:**
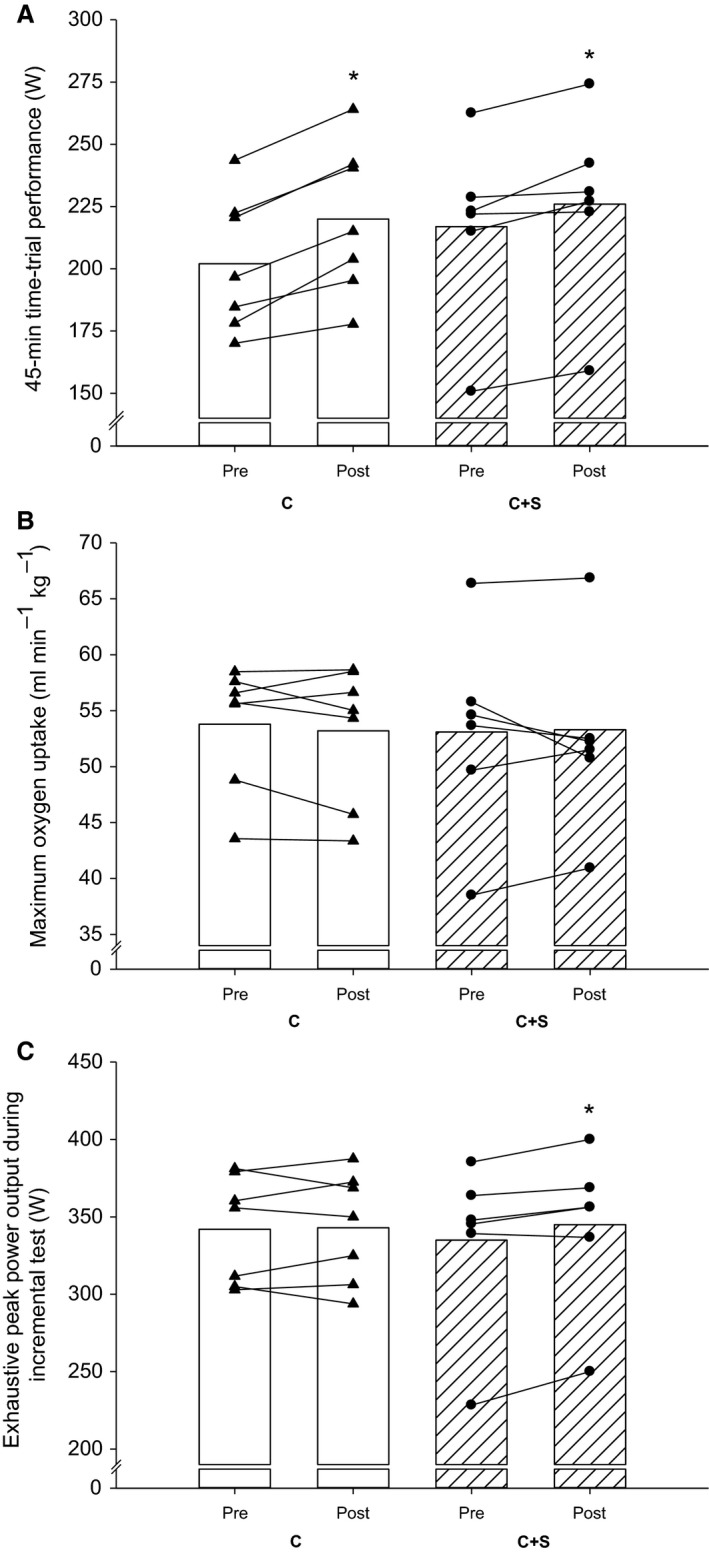
(A) 45‐min time‐trial performance (W), (B) maximum oxygen uptake, and (C) exhaustive peak power output (W) during incremental test before (pre) and after (post) an 8‐week intervention period consisting of 60 min of moderate intensity continuous cycling exercise without (C; white bars and black triangles; *n* = 7) or with (C+S; hatched bars and black circles; *n* = 6) inclusion of sprints. Mean and individual values are displayed. *Different (*P* < 0.05) from pre.

Performance in the TT improved (*P* < 0.001) by 4 ± 3 and 9 ± 3% with the intervention in C+S and C, respectively, with no difference between groups (Fig. 1A). No within‐ or between‐group changes were observed in $\dot{V}$O_2_‐max with the intervention (Fig. 1B). Peak power output achieved during an incremental test to exhaustion improved (*P* < 0.05) by 3 ± 3% with the intervention in C+S, whereas no change was observed in C (Fig. 1C), with no difference between groups.

### Heart rate and pulmonary oxygen consumption during the first and last training session

Average and peak heart rate were lower (*P* < 0.05) during the last training session compared to the first in both groups. Peak heart rate was higher (*P* < 0.01) during the first training session in C+S than in C (181 ± 6 vs. 169 ± 13 beats·min^−1^), whereas no difference in average heart rate was observed (152 ± 8 vs. 151 ± 13 beats·min^−1^). Pulmonary oxygen consumption was not different between groups during the first and last training session and no difference was observed within groups.

### Pulmonary gas exchange during submaximal cycling

No changes were observed in pulmonary gas exchange variables during submaximal cycling neither before nor after the intervention period in either group (Table 3).

**Table 3:** Oxygen uptake ($\dot{V}$O_2_), respiratory exchange ratio (RER), ventilation (V_E_), and V_E_/$\dot{V}$O_2_ during 5 min of submaximal cycling at 125 and 200W before (Pre) and after (Post) an 8‐week intervention period consisting of 60 min of continuous moderate intensity cycling exercise without (C; *n* = 7) or with (C+S; *n* = 6) inclusion of sprints

		C		C+S	
		Pre	Post	Pre	Post
$\dot{V}$O_2_ (L·min^−1^)	125 W	2.06 ± 0.22	2.02 ± 0.27	2.13 ± 0.07	2.14 ± 0.10
	200 W	3.03 ± 0.22	2.98 ± 0.27	3.13 ± 0.19	3.00 ± 0.10
RER	125 W	0.93 ± 0.05	0.93 ± 0.02	0.96 ± 0.10	0.93 ± 0.05
	200 W	0.98 ± 0.03	0.98 ± 0.03	1.02 ± 0.11	0.99 ± 0.06
V_E_ (L·min^−1^)	125 W	48.2 ± 6.1	48.5 ± 8.2	54.4 ± 8.2	52.4 ± 7.0
	200 W	76.2 ± 9.3	78.1 ± 13.4	89.3 ± 21.3	80.2 ± 12.1
V_E_/$\dot{V}$O_2_	125 W	23.4 ± 1.8	24.0 ± 1.3	25.5 ± 3.6	24.5 ± 2.2
	200 W	25.1 ± 2.4	26.2 ± 2.7	28.5 ± 6.0	26.7 ± 3.2

Values are presented as means ± SD.

### Blood variables during the first and last training session

**Figure 2:**
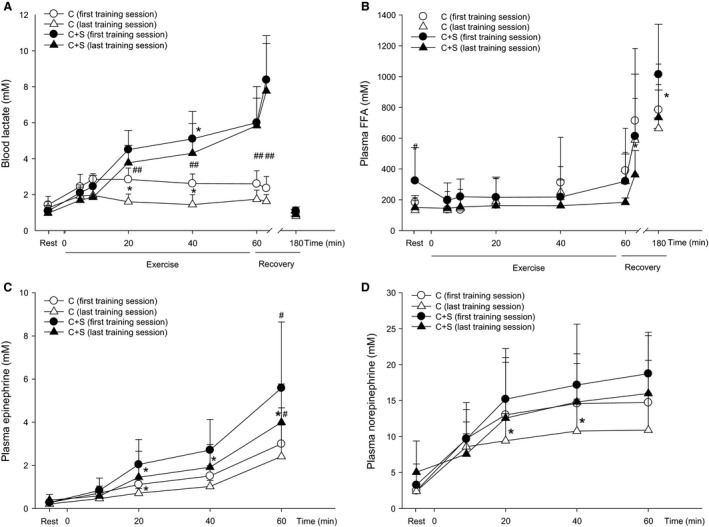
(A) blood lactate, (B) plasma FFA, (C) plasma epinephrine, and (D) plasma norepinephrine during the first (circles) and last (triangles) training session of an 8‐week intervention period consisting of 60 min of moderate intensity continuous cycling exercise without (C; white symbols; *n* = 7) or with (C+S; black symbols; *n* = 6) inclusion of sprints. Values are expressed as means±SD. *Different (*P* < 0.05) from first training session. ^#^Different (*P* > 0.05) from same time point in C.

Blood lactate was higher (*P* < 0.01) in C+S than in C at 20, 40, and 60 min, as well as 3 min after exercise during both the first and last training session (Fig. 2A). Blood lactate was lower (*P* < 0.05) at 40 min in C+S and at 20 and 40 min in C (Fig. 2A) during the last compared to the first training session.

Plasma FFA was higher (*P* < 0.05) in C+S than in C before the first training session (Fig. 2B). No within‐ or between‐group differences were observed in plasma FFA during exercise (Fig. 2B) from the first to the last training session. In C+S, plasma FFA was lower (*P* < 0.05) in recovery from exercise after the last compared to the first training session, whereas no difference was observed in C (Fig. 2B).

At the end of exercise, plasma epinephrine was higher (*P* < 0.05) in C+S than in C during the first and last training session, with no difference between groups in plasma norepinephrine. Plasma epinephrine was lower (*P* < 0.05) after the intervention at 20, 40, and 60 min in C+S and at 20 min in C. After the training intervention, plasma norepinephrine during exercise was lower (*P* < 0.05) at 20 and 40 min in C with no difference in C+S (Fig 2C+D).

### Muscle metabolic response during the first and last training session

Muscle PCr content declined (*P* < 0.01) during the first and last training session in both groups, with a larger decline in C+S than in C both before and after the intervention (Table 4). No change in muscle PCr degradation was observed from the first to the last training session within groups (Table 4).

**Table 4:** Muscle creatine phosphate (PCr), lactate, glycogen, and pH before (0 min) and immediately after (60 min) the first and last training session of an 8‐week intervention period consisting of 60 min of continuous moderate intensity cycling exercise without (C; *n* = 6) or with (C+S; *n* = 6) inclusion of sprints

	Time (min)	C		C+S	
		First	Last	First	Last
PCr (mmol·kg dw^−1^)	0	84.0 ± 20.0	79.3 ± 9.4	83.3 ± 9.1	78.1 ± 9.4
	60	50.5 ± 15.1†	51.3 ± 16.1†	38.7 ± 15.6†#	26.1 ± 3.2†#
Lactate (mmol·kg dw^−1^)	0	16.0 ± 3.4	16.3 ± 8.3	12.3 ± 7.6	9.8 ± 5.4
	60	24.6 ± 7.3	12.9 ± 3.0*	72.8 ± 16.5†#	76.1 ± 19.2†#
Glycogen (mmol·kg dw^−1^)	0	599 ± 137	712 ± 215	528 ± 109	657 ± 87
	60	374 ± 167†	524 ± 190†	238 ± 157†	329 ± 86†
pH	0	7.15 ± 0.07	7.19 ± 0.09	7.18 ± 0.08	7.16 ± 0.10
	60	7.12 ± 0.06	7.16 ± 0.06	6.63 ± 0.42†#	6.96 ± 0.13*†#

Values are presented as means±SD. *Different (*P* < 0.05) from first training session. ^†^Different (*P* < 0.05) from rest. ^#^Different (*P* < 0.05) from C.

Muscle lactate increased (*P* < 0.001) more in C+S than in C during both the first and last training session, with no change in C+S, but a lower (*P* < 0.05) muscle lactate accumulation during the last compared to the first training session in C (Table 4).

Muscle pH was lower (*P* < 0.05) in C+S than in C immediately after both the first and last training session (Table 4). In C+S, muscle pH was higher (*P* < 0.01) immediately after the last compared to the first training session, whereas no difference was observed in C (Table 4).

After the training intervention, muscle glycogen at rest was nonsignificantly higher (*P* < 0.10) in both groups whereas muscle glycogen breakdown was the same in the first and last training session in both groups (Table 4).

### mRNA expression of PGC‐1α

**Figure 3:**
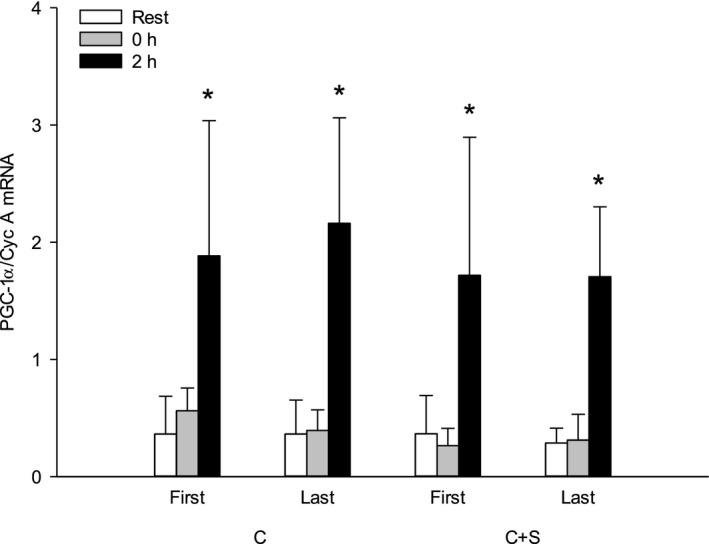
Peroxisome proliferator‐activated receptor‐ϒ coactivator‐1*α* (PGC‐1*α*) mRNA before (rest; white bars), immediately (gray bars) and 2 h (black bars) after 60 min of moderate intensity continuous cycling exercise without (C; white bars; *n* = 6) or with (C+S; black bars; *n* = 6) inclusion of sprints, before and after an 8‐week intervention period. Values are expressed as means±SD. *Different (*P* < 0.01) from before (rest) and immediately after (0 h) exercise.

Two hours after the first training session, PGC‐1*α* mRNA was ~4‐ and ~5‐fold higher than at rest in C+S and C, respectively. No within‐ or between‐group changes were observed in the exercise‐induced PGC‐1*α* mRNA with the intervention (Fig. 3A).

### Protein expression

**Figure 4:**
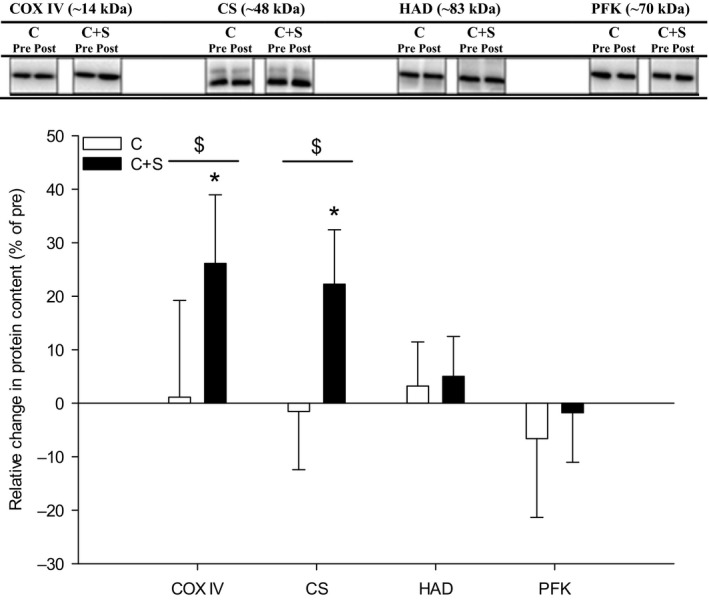
Relative change in muscle protein expression after an 8‐week intervention period consisting of 60 min of moderate intensity continuous cycling exercise without (C; white bars; *n* = 6) or with (C+S; black bars; *n* = 6) inclusion of sprints. Values are expressed as means±SD. *Different (*P* < 0.05) from before the intervention period. $Group x time interaction effect (*P* < 0.05). Cytochrome C Oxidase complex IV (COX IV), *β*‐hydroxyacyl‐CoA dehydrogenase (HAD), citrate synthase (CS), and phosphofructokinase (PFK).

**Figure 5:**
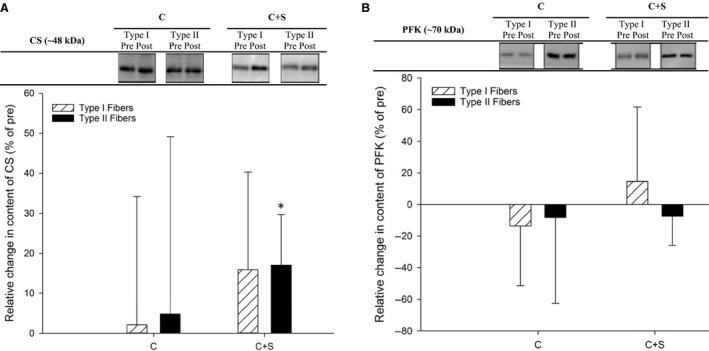
Relative change in muscle protein expression of (A) citrate synthase and (B) phosphofructokinase in type I (cross‐hatched bars) and type II (hatched bars) single muscle fibers after an 8‐week intervention period consisting of 60 min of moderate intensity continuous cycling exercise without (C; *n* = 6) or with (C+S; *n* = 6) inclusion of sprints. Values are expressed as means ± SD. *Different (*P* < 0.05) from before the intervention period.

Expression of whole muscle COX IV and CS increased (*P* < 0.05) with the intervention in C+S, whereas no change was observed in C (Fig. 4), with the changes being greater (COX IV: *P* < 0.05 and CS: *P* < 0.05) in C+S than C. In type II fibers, content of CS increased (*P* < 0.05) with the intervention in C+S, with no change in type I fibers. In C, no change in CS content in neither type I nor type II fibers was observed with the intervention (Fig. 5A).

Content of whole muscle HAD and PFK content did not change with the intervention in either group (Fig. 4). No change in content of HAD and PFK was observed in type I and type II fibers, respectively, within‐ or between‐ groups (Fig. 5B).

### Maximal muscle enzyme activity

No change in maximal activity of HAD and CS was observed within or between groups (Table 5). Maximal activity of PFK did not change within groups, but the change in maximal activity of PFK in C+S was higher (*P* < 0.05) than in C.

**Table 5:** Maximal activity of muscle phosphofructokinase (PFK), hydroxyacyl‐CoA dehydrogenase (HAD), citrate synthase (CS), and pH before (Pre) and after (Post) an 8‐week intervention period consisting of 60 min of continuous moderate intensity cycling exercise without (C; *n* = 6) or with (C+S; *n* = 6) inclusion of sprints

	C		C+S	
	Pre	Post	Pre	Post
PFK (*μ*mol·g dw^−1^ min^−1^)	377 ± 63	336 ± 48	355 ± 89	368 ± 104
HAD (*μ*mol·g dw^−1^ min^−1^)	18.9 ± 2.4	17.7 ± 2.1	18.9 ± 4.4	21.6 ± 4.0
CS (*μ*mol·g dw^−1^ min^−1^)	37 ± 14	35 ± 15	41 ± 8	42 ± 8

Values are presented as means ± SD.

## Discussion

The major findings of this study were that addition of 30‐s sprints in moderate intensity continuous training led to superior improvements in whole muscle content of COX IV and CS in trained males compared to work‐ and duration‐matched moderate intensity continuous training. The different response occurred despite a similar exercise‐induced increase in the PGC‐1*α* mRNA levels during the first and last training session between the two groups.

The higher content of CS and COX IV in the group including sprints may have been due to higher muscle metabolic perturbations during sprints. During the first and last training session muscle and blood lactate concentrations were higher and muscle PCr and pH lower in the group including sprints. The lower muscle PCr suggests a lower intracellular energy state, which may have activated intramuscular sensors such as AMPK, p38 MAPK, and the specific transcription factor CREB, which are key components in signaling cascades inducing mitochondrial biogenesis in human skeletal muscle (Puigserver et al. 2001; Jager et al. 2007; Egan and Zierath 2013). In support, phosphorylation of p38 MAPK and CREB was elevated more following a moderate intensity continuous exercise protocol including six repeated 30‐s sprints compared to work‐ and duration‐matched moderate intensity continuous exercise in trained males (Brandt et al. 2016).

The plasma epinephrine concentrations were higher during exercise in the group including sprints, which may have contributed to the increase in muscle oxidative enzymes. In support, *β*‐adrenoceptor blockade during endurance exercise blunts training‐induced increases in maximal activity of oxidative enzymes (Svedenhag et al. 1984; Wolfel et al. 1986) and injection of epinephrine or clenbuterol (a beta_2_‐adrenergic agonist) has been shown to increase PGC‐1*α* mRNA in mouse skeletal muscle (Miura et al. 2007; Chinsomboon et al. 2009) suggesting that circulating epinephrine levels may play a role in mitochondrial biogenesis. On the other hand, we recently demonstrated a lack of association between plasma epinephrine levels and exercise‐induced PGC‐1*α* mRNA response (Brandt et al. 2016), and that beta_2_‐adrenergic agonist attenuated adaptations in $\dot{V}$O_2_‐max and oxidative enzymes induced by endurance training (Hostrup et al. 2018). Thus, circulating epinephrine levels do not appear to influence early exercise‐induced transcription of mitochondrial proteins in trained human skeletal muscle.

Citrate synthase content in type II muscle fibers was elevated only in the group including sprints, which may be due to a greater recruitment of type II fibers during sprints (Krustrup et al. 2004). We observed no change in CS content in type I fibers, which is in line with recent observations in trained individuals after a period of sprint training (Christensen et al. 2015; Nyberg et al. 2016; Skovgaard et al. 2018) and may be related to larger abundance in type I than type II muscle fibers (Howald et al. 1985; Thomassen et al. 2013; Christensen et al. 2015), this is however speculative. The level of muscle oxidative enzymes did not change in the moderate intensity continuous training group which is in agreement with observations of well‐trained subjects training for 30‐60 min per session (Bangsbo et al. 2009; Iaia et al. 2009; Gunnarsson and Bangsbo 2012). In contrast, studies of untrained have demonstrated marked increases in muscle oxidative enzymes (Gollnick et al. 1973; Saltin et al. 1976; Gollnick and Saltin 1982). Thus, the unchanged level of oxidative enzymes observed following the moderate intensity continuous training group may be related to the training status of the subjects (~54 mL·kg^−1^·min^−1^).

The increase in PGC‐1*α* mRNA levels with exercise in this study (~4–5 fold) was similar to what has been reported after 4 × 30‐s all‐out sprints (Gibala et al. 2009; Cochran et al. 2014), but lower than the 10‐ to 12‐fold increase reported after moderate intensity continuous (Stepto et al. 2012) and HIIT (Perry et al. 2010) exercise. The contrasting findings may be explained by differences in training status, as the PGC‐1*α* mRNA response has been shown to be affected by training status (Nordsborg et al. 2010). Thus, subjects (in this study) had ~25% higher $\dot{V}$O_2_‐max compared to the aforementioned studies (Perry et al. 2010; Stepto et al. 2012), which may, in part, explain the lower exercise‐induced PGC‐1*α* mRNA response. In support, Psilander et al. (2010) found similar increases in PGC‐1*α* mRNA response (~6‐fold) in well‐trained cyclists ($\dot{V}$O_2_‐max ~68 mL·kg^−1^·min^−1^) following moderate intensity continuous (3*20 min at 87% of $\dot{V}$O_2_‐max) and sprint (7 × 30‐s all‐out sprints interspersed by 4.5 min of recovery) exercise, suggesting of a blunted muscle PGC‐1*α* mRNA response following moderate intensity continuous exercise in trained compared to untrained muscle. Nevertheless, despite the similar increase in PGC‐1*α* mRNA within groups during both the first and last training session, a higher expression of muscle oxidative enzymes was observed only in the group including sprints. These findings suggest that elevated PGC‐1*α* mRNA levels do not necessarily lead to mitochondrial biogenesis in trained muscle. Correspondingly, studies in mice have shown that PGC‐1*α* is not mandatory for training‐induced adaptations in oxidative proteins (Leick et al. 2008). Thus, the mRNA response to acute exercise may not accurately predict skeletal muscle adaptations to exercise training (Cochran et al., 2014) suggesting that mRNA transcription has to be recognized as presenting only the first step toward protein synthesis, and the assessment of post‐transcriptional regulating mechanisms (i.e., microRNAs) may improve our understanding of molecular events originating from exercise‐induced muscular adaptations (Miller et al. 2016). Nonetheless, our findings are supported by studies showing that sprint training promotes mitochondrial biogenesis in untrained skeletal muscle (Jacobs et al. 1987; MacDougall et al. 1998; Burgomaster et al. 2006; Gibala et al. 2006). However, more studies are needed to elucidate the underlying mechanisms in mitochondrial adaptations between work‐ and duration‐matched exercise protocols as employed in this study.

We observed no difference in the PGC‐1*α* mRNA response from the first to the last training session between groups. This was unexpected as exercise has been shown to amend the exercise‐induced PGC‐1*α* mRNA levels following a training period (Pilegaard et al. 2003; Perry et al. 2010). The similar exercise‐induced PGC‐1*α* mRNA response within and between protocols may be explained by differences in training status (Nordsborg et al. 2010) training duration, and/or timing of muscle biopsies (Pilegaard et al. 2003). Subjects in this study were trained and muscle biopsies were taken 2 h after cessation of exercise, but the influence of these factors on the exercise‐induced PGC‐1*α* mRNA response is not well understood and further studies investigating the effect of each of these variables on the exercise‐induced PGC‐1*α* mRNA response and muscle adaptations are warranted. Thus, we cannot exclude that the PGC‐1*α* protein has been elevated more when sprints are included in the moderate intensity continuous exercise training, as PGC‐1*α* protein content has been reported to be regulated by post translational modifications (Puigserver et al. 2001; Jager et al. 2007). This remains, however, unknown but could in part explain the different adaptations in oxidative enzymes between groups following moderate intensity continuous exercise training with or without inclusion of sprints.

Time‐trial performance improved to the same extent between groups after the intervention despite an increase in the content of oxidative enzymes in the group including sprints. The similar improvement in TT performance was not caused by changes in $\dot{V}$O_2_‐max or cycling efficiency, as they did not change in either group. In the moderate intensity continuous training group, the better TT performance may be explained by a higher relative work intensity after the training intervention, reflected in lower muscle and blood lactate during the last compared to the first training session. This observation fits well with the lower levels of plasma catecholamines observed during the last training session, as catecholamines are major effectors of lactate production through modulation of muscle glycogenolysis (Duan and Winder 1994). It is widely accepted, that the exercise intensity corresponding to the lactate threshold is a powerful predictor of endurance performance (Coyle et al. 1991; Jones and Doust 1998) and a right shift in this variable, as observed with exercise in the moderate intensity continuous training group, is highly likely to improve TT performance.

The improved TT performance in the group including sprints may have been due to a higher anaerobic capacity, as performance during the incremental test to exhaustion improved without an increase in $\dot{V}$O_2_‐max indicative of an improved anaerobic capacity. In support, the marked reduction in muscle pH at the same lactate accumulation suggests that muscle H^+^ transport and/or intracellular muscle buffering was improved in this group. In accordance, an improved in vivo buffer capacity has been observed in trained cyclists following 8 weeks of high‐intensity training (Gunnarsson et al. 2013). The observation that the TT performance improved similarly between groups, despite a superior increase in whole muscle COX IV and CS content in the group including sprints, indicates that the level of oxidative enzymes is not essential for changes in endurance performance in trained individuals. In agreement, endurance exercise performance has been shown to improve following a period of intense training in trained individuals, despite no change in content or activity of oxidative enzymes (Bangsbo et al. 2009; Gunnarsson and Bangsbo 2012; Gunnarsson et al. 2013; Skovgaard et al. 2014).

In summary, that muscle COX IV and CS protein content increased more after moderate intensity continuous exercise training with inclusion of sprints, despite similar exercise‐induced PGC‐1*α* mRNA responses after the first and last training session within and between groups, suggest that the exercise‐induced PGC‐1*α* mRNA level is not predictive of changes in muscle mitochondrial adaptations in trained individuals. The increase of COX IV and CS in whole muscle and CS in type II muscle fibers in the group including sprints, may have been caused by greater muscle metabolic disturbances as well as higher plasma epinephrine levels during exercise. In addition, that TT performance improved similarly between groups, despite different mitochondrial adaptations; suggest that changes in mitochondrial adaptations may be of less importance for improvements in endurance performance in trained males, whether or not sprints are included in work‐ and duration‐matched moderate intensity continuous exercise training.

## Conflict of Interest

No conflict of interests.
